# Fabrication, Characterization and Photocatalytic Activity of Copper Oxide Nanowires Formed by Anodization of Copper Foams

**DOI:** 10.3390/ma14175030

**Published:** 2021-09-02

**Authors:** Alaa M. Abd-Elnaiem, Moustafa A. Abdel-Rahim, Atta Y. Abdel-Latief, Ahmed Abdel-Rahim Mohamed, Kristina Mojsilović, Wojciech Jerzy Stępniowski

**Affiliations:** 1 Physics Department, Faculty of Science, Assiut University, Assiut 71516, Egypt; mabdelrahim@aun.edu.eg (M.A.A.-R.); attayousef@aun.edu.eg (A.Y.A.-L.); ahmed.201297@science.au.edu.eg (A.A.-R.M.); 2 Academy of Scientific Research and Technology (ASRT) of the Arab Republic of Egypt, Cairo 11516, Egypt; 3 Faculty of Physics, University of Belgrade, Studentski trg 12–16, 11000 Belgrade, Serbia; kristina.mojsilovic@ff.bg.ac.rs; 4 Faculty of Advanced Technology and Chemistry, Institute of Materials Science & Engineering, Military University of Technology, Kaliskiego 2 Str., 00908 Warszawa, Poland

**Keywords:** copper oxides, anodization, nanowires, FTIR, photocatalytic decolorization, nanostructures, tenorite, cuprite, paramelconite, malachite

## Abstract

In recent paper anodization of copper foams in 0.1 M K_2_CO_3_ is reported. Anodization was performed in the voltage range of 5–25 V and in all the cases oxides with a developed surface area were obtained. However, anodizing only at 20 and 25 V resulted in the formation of nanostruc-tures. In all the cases, the products of anodizing consisted of crystalline phases like cuprite (Cu_2_O), tenorite (CuO), parameconite (Cu_4_O_3_) as well as spertiniite (Cu(OH)_2_). Copper foams after ano-dizing were applied as catalysts in the photocatalytic decolorization of a model organic compound such as methylene blue. The highest photocatalytic activity was observed for samples anodized at 25 V and closely followed by samples anodized at 5 V. The anodized copper foams proved to be a useful material in enhancing the photocatalytic efficiency of organic dye decomposition.

## 1. Introduction

Recent emerging issues such as water purification, filtration, and materials for renewable energy harvesting and storage, as well as carbon dioxide reduction, increase demand for novel nanostructured materials [1]. Research on the nanostructures materials will have an invaluable impact on the progress in catalysts performance, device miniaturization, and assembly or energy harvesting and storage [2]. Nanostructures, such as nanorods, nanowires, and nanotubes made of metallic and semiconducting materials, have attracted much interest because of their technological applications in different fields such as nanoelectronics, optoelectronics, sensors, energy, and magnetic storage [3].

One of the key materials that served as a template in these nanostructures fabrication is porous anodic alumina (PAA) [2,3,4,5,6,7,8]. Porous anodic alumina is formed by electrochemical oxidation of aluminum that results in the formation of porous oxide. According to Masuda and Fukuda’s milestone paper, a two-step self-organized process results in the formation of a well-ordered honeycomb made of alumina [5]. Since it was found that the morphology of the nanoporous anodic alumina can be fully controlled by the operating conditions, such as the type of electrolyte, applied voltage, temperature, and duration of the process, it has triggered an incredible amount of research and reports on the formation of the nanostructures [5]. The features such as high surface area and control of the pores morphology of anodic alumina allowed achieving templates in a much more efficient way compared to the polymer templates [6]. The PAA templates can then be used for various fabrication of nanostructures, including the nanowires, nanodots, and nanotubes of various materials [7,8].

Anodization of aluminum with the formation of nanostructures triggered research on the electrochemical oxidation of other metals such as titanium, iron, hafnium, tungsten, zirconium, zinc, etc., [2,3,4,5,6,7,8]. In general, the majority of the anodic oxides have fixed stoichiometry, are amorphous, and are made of nanopores or nanotubes. When compared to them, anodic copper oxides seem to be unique—these have non-fixed stoichiometry (made simultaneously of cuprous oxide, cupric oxide, and cupric hydroxide), consist of nanowires or nanorods, and are crystalline: cuprous oxide crystallizes as regular cuprite, while cupric oxide crystallizes as a monoclinic tenorite [9,10,11,12,13]. Anantharaj et al. recently presented a potentiostatic method of anodization of copper foil for a shorter amount of time in a three-electrode system to form a tall (5–7 µm) nanoneedle array of copper oxide [14,15]. The proposed method showed a better time and energy efficiency and concluded that the anodization time is inversely proportional to the pH of the electrolyte in which anodization is being carried out. Moreover, it revealed that for high pH, extremely short anodizings are sufficient to obtain nanostructures on copper.

According to the physical properties, copper oxides, e.g., cupric oxide (CuO) and cuprous oxide (Cu_2_O), are similar to most of the transition metal oxides in terms of their band structure and photocatalytic performance, low thermal emittance, non-toxicity, and simple and low-cost synthesis [16,17]. It is generally known that CuO is a p-type semiconductor with a bandgap of 1.2–1.7 eV, and hence it is possible to absorb light in the visible spectra region [18]. Besides the photocatalytic activity of copper oxides, it can also be used for highly effective hydrogen generation by water splitting when combined with titanium dioxide [19]. Copper oxides can easily be formed by the thermal oxidation of copper at elevated temperatures [20]. Recently, nanostructured copper oxides such as nanowires for various technological applications have been prepared [21,22,23,24].

One of the recent novel methods is the formation of copper oxide nanostructures by anodizing. This self-organized process allows to form mixed Cu_2_O-CuO system with highly-developed surface area, demanded in fields such as catalysis and sensing. In this paper, the formation of copper oxide nanowires by anodizing and their application in photocatalytic decolorization of methylene blue as a model dye is reported. The ultimate goal of the presented research is to find relations between operating conditions, material features, mainly composition and morphology, and kinetics of photocatalytic decolorization. What is even more important, up to date, only individual papers are focused on anodizing copper electrodes that are not planar. So far, only Xu et al. reported anodizing of copper mesh [21]. In this paper, we report, for the first time, successful anodization of copper foam, its characterization, and application as a photocatalyst. The developed surface area of copper foam, enhanced by electrochemical nanostructuring, enables new possibilities in emerging applications.

## 2. Materials and Methods

A high-purity copper foam (>99.99%) was purchased from Suzhou Jiashide metal foam Co. Ltd, Xiamen, China, and cut into rectangular coupons of 25 mm × 6 mm. The anodization of copper foams was performed at room temperature (30 °C) in 0.1 M K_2_CO_3_ at various anodizing voltages in the range of 5–25 V. The anodization was conducted in a two-electrode cell setup. The working electrode is the copper foam, while the counter electrode that is used is the platinum wire electrode. The anodizing voltage and response current density during the anodization were recorded using PGSTAT302N Autolab system Metrohm (Herisau, Switzerland). Each anodization was performed for 5 h, and the exposed area of the samples was limited to 10 mm × 6 mm for each side.

Characterization of the obtained materials was performed with series of techniques. Fourier Transformed Infra-Red Spectroscopy (FTIR; Termo-Nicolet-6700 FT-IR) was applied to investigate the chemical composition of the obtained oxides. The FTIR spectra were recorded in the 4000–400 cm^−1^ wavenumber range on solid samples by making a pellet with KBr. X-ray diffraction experiments were performed to check the crystalline phases that the products of anodizing contained. The CuKα radiation (λ = 1.546 Å) was used in the X-ray (Bruker, D8 advanced Cu target, Karlsruhe, Germany) to study the crystal structure. The data were cured in the 2θ range of 20–70° with a rate of 0.06°/s. Field emission scanning electron microscopy (FE-SEM) provided important data on the morphology of the nanostructures. The Zeiss Sigma 500 VP analytical FE-SEM, Zeiss-SIGMA Company, Zeiss, Germany, was used, and imaging using SE detector was performed.

For photocatalysis, 0.01 g of every anodized copper sample was taken, suspended in 25 mL of 10^−5^ M of Methylene Blue (MB). We put the five samples in dark mode for equilibrium achievement and measured the absorbance without irradiation. We exposed another five samples to the irradiation at different times and measured the absorbance every 0, 20, 60, 100, 140, 260, and 380 min. The absorbance was measured using Perkin Elmer 750 Lambda at the analytical wavelength equal to 200–900 nm at room temperature. The experiments were carried out in a Pyrex reactor and an artificial Sunlight Simulator of 1800 W (Model Oriel SO12A) associated with an ultraviolet filter. The actually received intensity on the catalysts samples was evaluated to be 100 mW/cm^2^. The decolorization of organic dye was monitored by measuring the changes in the UV-Vis absorbance spectra as a function of irradiation time. The spectra were measured using Lambda 750 UV-Vis spectrophotometer (PerkinElmer, UK) with a scanning rate of 3 nm/s.

## 3. Results & Discussion

**Figure 1:**
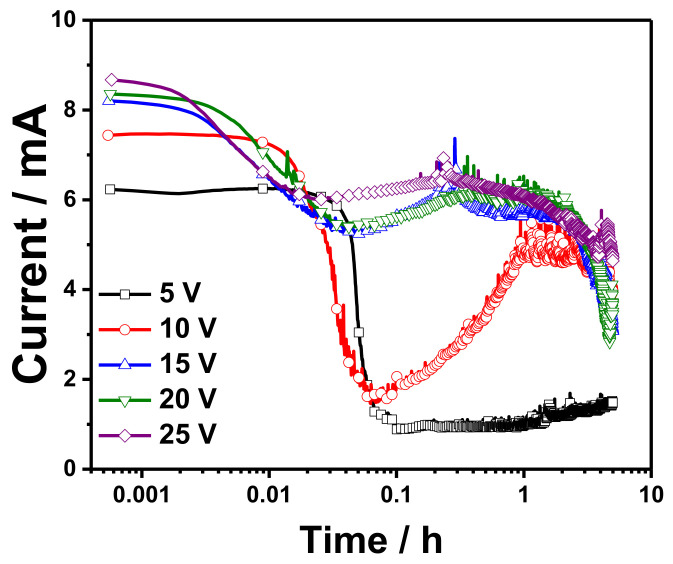
Current-time curves recorded during the anodization of copper foam in 0.1 M K_2_CO_3_ at 5, 10, 15, 20, 25, and 25 V.

Figure 1 shows the current-time (*i*-t) curves recorded during the potentiostatic anodization of copper foam in 0.1 M K_2_CO_3_ at 5, 10, 15, 20, and 25 V. The anodization process was performed at room temperature (~30 °C) for 5 h.

As the anodization of copper starts, the copper foam quickly transforms to light blue color, and after long anodization, the anodized part transforms to a dark blue color which indicates the formation of copper (II) hydroxide and copper (II) oxide, respectively. Additionally, some oxygen bubbles were noted at the copper foam (anode) and could be linked to the oxidation of H_2_O and OH^–^ to oxygen (Oxygen Evolution Reaction, OER). The formed oxygen bubbles could be a reason for the observed low current during the anodization process. The trends of all the transient curves except 5 V are similar to the anodization of aluminum foils, as was reported in previous research [11]. The transient curve mainly formed from three sections followed three different processes that occur during the anodization of copper foam. In the first stage, the current rapidly drops from a high to the lowest value. The duration of this stage is 5.8, 3.8, 1.9, 2.5, and 3.3 min for the anodization performed at 5, 10, 15, 20, and 25 V, respectively. This corresponds to the formation of an oxide layer along the whole surface, increasing the electric resistance of the whole circuit. Copper oxides are semiconductors, not insulators, so the change is not so dramatic as in the case of aluminum anodizing. Nevertheless, it is apparent that there is a rapid drop in current caused by the chemical reactions and morphological changes at the electrode–electrolyte interface. The observed minimum current in this stage reveals that the adsorption and nucleation are comparable, and the nucleation and growth of the film become the dominant component of current. In the second stage, the current starts to increase to a maximum value to reach the *plateau* region (the third stage). The observed maximum reveals that the nucleation of the passive film is faster than the adsorption process. Moreover, it indicates that the surface area of the oxide has been developed. The observed stages in the *i*-t curves reveal that the growth of the passive oxides takes place according to the adsorption–nucleation mechanism [24]. The adsorption–nucleation mechanism shows that the current comes from two major phenomena: rapid decrease of the current, indicating adsorption of the ions at the working electrode, and current rise, depicting nucleation. The second phenomenon is attributed to the two-dimensional growth of anodic oxide governed by diffusion. Similar observations were observed for the anodization of copper foil in sodium hydroxide obtained at −0.05 mV to −0.2 mV [13]. What is also worth noticing, at the steady-state growth of the oxide, the current drops gradually. It indicates that during the anodizing, the oxide growth is resulting in increased electric resistance, depicted in Figure 1 as a gradual, slow current decrease.

**Figure 2:**
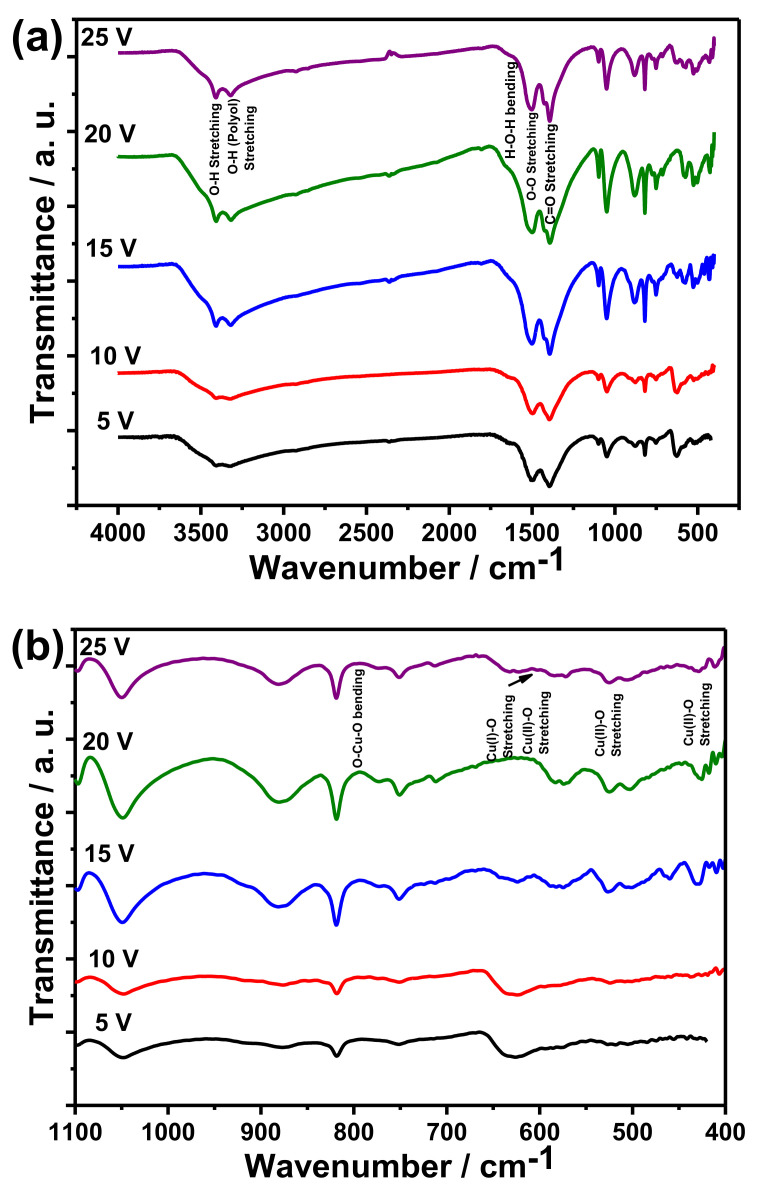
(**a**) FTIR spectra of the anodized copper foam in 0.1 M K_2_CO_3_ at 5, 10, 15, 20, 25, and 25 V; and (**b**) enlarged FTIR spectra in the range of 400–1100 cm^−1^.

FTIR spectra provide information on the chemical composition of the grown oxide. Figure 2 shows the FTIR spectra for the anodized copper foams at various voltages. The observed FTIR peaks (Figure 2) confirm the presence of CuO and Cu_2_O. The observed peaks at 432, 530, and 606 cm^−1^ belong to the Cu(II)-O bond, while observed peaks at 624 cm^−1^ are assigned to the F_1u_ modes of the Cu_2_O phase [25]. The FTIR peak at 624 cm^−1^ corresponds to the characteristic stretching vibration of the Cu (I) bond in the Cu_2_O phase. The observed peaks at 432, 530, and 606 cm^−1^ can be assigned to the A_u_ mode, B_u_ mode, and the other B_u_ mode of CuO, respectively. These FTIR peaks correspond to characteristic stretching vibrations of the Cu(II)-O bond in the CuO phase. The band at 1384 cm^−1^ was ascribed to the C=O bond. It reveals that probably carbonates from the electrolyte were incorporated into the grown oxide. The absorption band at 1623 cm^−1^ and 3478 cm^−1^ was ascribed to H–O–H bending and O–H stretching, respectively, which means that they formed oxides that can be partially hydrated. The formation of O_2_ ads on CuO was confirmed, judging from the band of υ(O–O) at around 1500 cm^−1^ [26]. The change of intensity of the same peaks as the anodizing voltage could be attributed to the change in the crystallinity, as the intensity could be increased when the crystallinity improved. The observed bands at 886 cm^−1^ and 3398 cm^−1^ belong to the tocking vibrational mode and stretching vibration of the water molecule, respectively. The seen bands of 3328 cm^−1^ were attributed to O–H (polyol) [27]. The observed peaks between 827 cm^−1^ and 821 cm^−1^ belonged to the bending vibrations of O-Cu-O.

**Figure 3:**
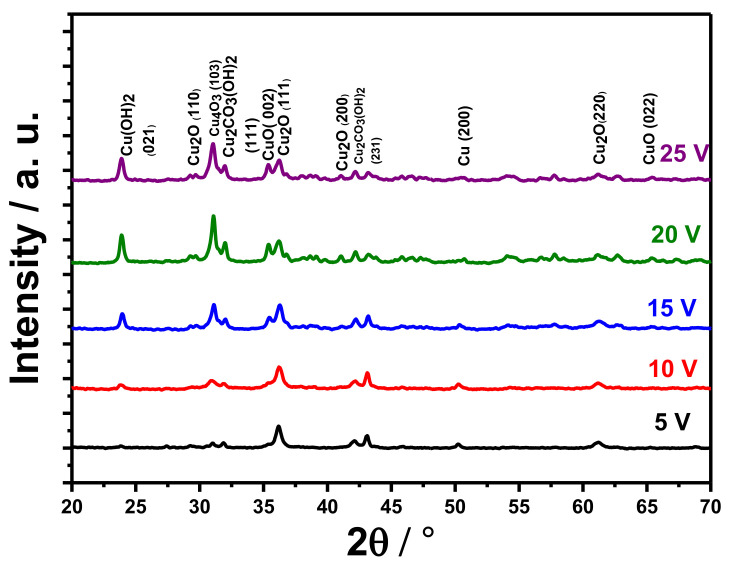
XRD patterns of the anodized copper foam in 0.1 M K_2_CO_3_ at 5, 10, 15, 20, 25, and 25 V.

X-ray diffraction patterns reveal that anodizing of copper foams results in the formation of crystalline oxidation products (Figure 3). There are distinct reflections confirming the presence of cubic cuprite, Cu_2_O, in the XRD pattern, namely from (110), (111), and (220) planes. Moreover, monoclinic tenorite, CuO, was also detected, and reflections from (002) and (022) planes were found to be distinct in the XRD pattern. The crystal structure, interplanar spacing (d), the average crystallite size (D), microstrain (ε), and dislocation density (δ) for various formed phases are summarized in Table 1. The crystal structure for Cu(OH)_2_, Cu_2_O, Cu_4_O_3_, Cu_2_O_3_(OH)_2_, and CuO is orthorhombic, cubic, tetragonal, monoclinic, and monoclinic, respectively.

In general, these findings are in agreement with the literature, where usually crystalline tenorite and cuprite are detected after Cu anodizing [9,10,11,12,13,28]. It is also important to note that, in particular cases, annealing after anodizing has to be applied to detect crystalline oxides, for example, copper electrochemical oxidation in 2 M KOH at 1.5 mA·cm^−2^.

This leads to the formation of Cu(OH)_2_, and annealing 3 h at 150 °C or 200 °C leads to hydroxide decomposition and formation of crystalline tenorite and cuprite [22]. Paramelconite, Cu_4_O_3_, an oxide with mixed oxidation states of copper, was also detected within the XRD experiments; however, reflex from only (103) plane was distinct in the XRD patterns. Detection of this oxide is quite unique when compared to other reports; nevertheless, it was reported to be obtained by copper anodizing in 0.1 M K_2_CO_3_ [9] and 0.01 M NaHCO_3_ [22].

Additionally, spertiniite, crystalline Cu(OH)_2_ was detected with XRD. Reflex from the (021) plane was detected. It is worth noting that in some cases, the anodically formed cupric hydroxide is amorphous; in other cases, it can be crystalline. Obtained results are in line with planar copper anodizing in 0.1 M K_2_CO_3_ [9] and 0.01 M NaHCO_3_ [22].

XRD patterns also confirm the incorporation of electrolyte anions into the formed products of anodizing. Reflexes from (111) and (221) planes of malachite, Cu_2_CO_3_(OH)_2_ were noticed, which is in line with previously reported research on planar copper anodizing in 0.1 M K_2_CO_3_ [9] and 0.01 M NaHCO_3_ [22]. Moreover, FTIR experiments also confirmed the presence of C-O bondings (Figure 2). This phenomenon is analogous with anions incorporation in other anodic oxides [29], with one exception: in the case of copper anodizing, crystalline compounds, such as malachite, are formed.

To sum up, electrochemical oxidation of copper foam allowed the formation of tenorite, cuprite, paramelconite, and spertiniite, accompanied by malachite. Therefore, this process allows the simultaneous formation of a variety of products, with various valence states on the surface, demanded in various types of catalysis.

**Figure 4:**
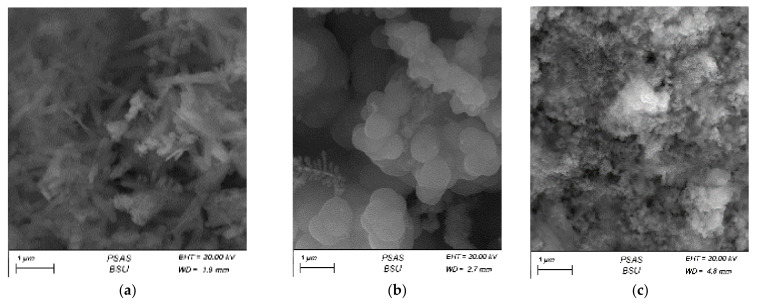

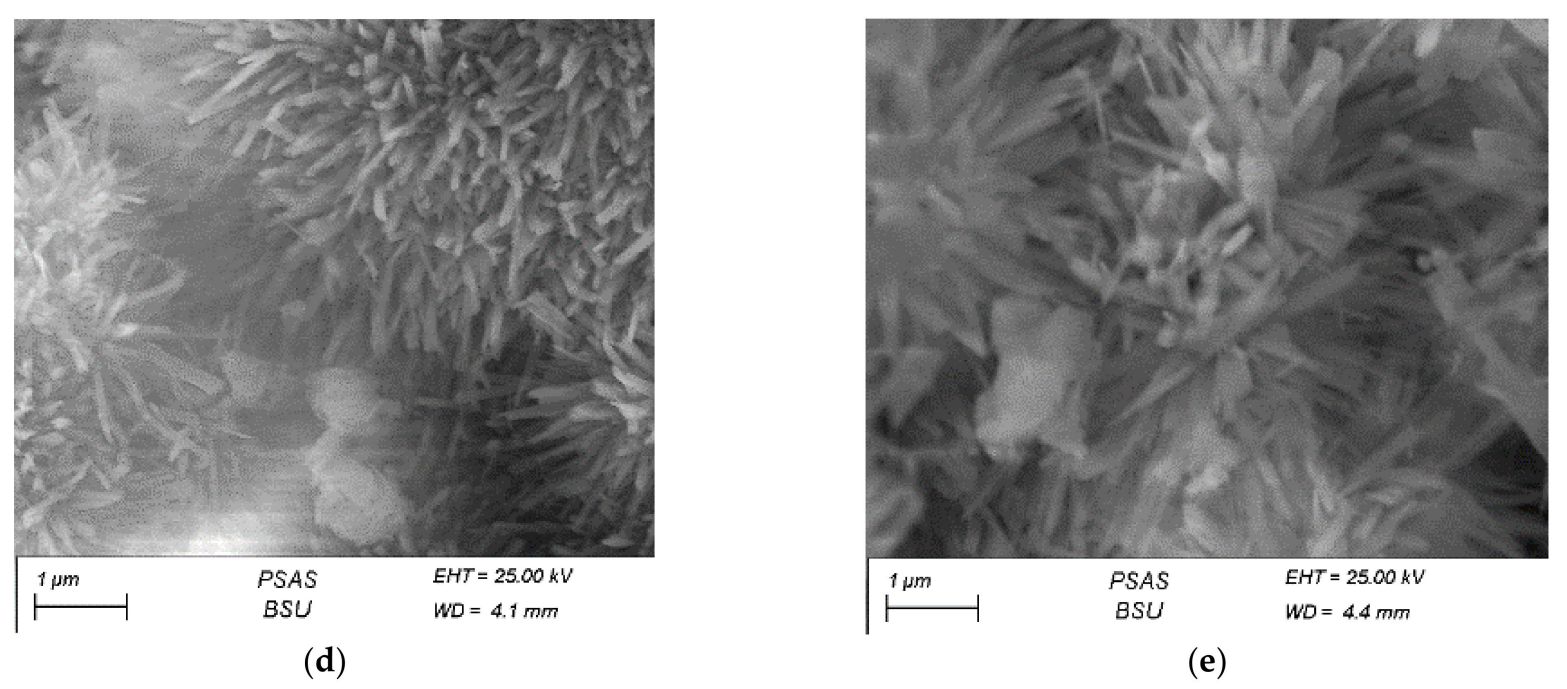
(**a**) SEM images of the anodized copper foam in 0.1 M K_2_CO_3_ at 5, (**b**) 10, (**c**) 15, (**d**) 20, and (**e**) 25 V (scale bare 1 µm).

Another feature important in catalysis is developed surface area. FE-SEM imaging reveals that for Cu foam anodizing at relatively low voltages, namely 5–15 V formed oxides developed surface area, but nanostructures were not found (Figure 4). Since 20 V, nanowires were successfully formed. Analogous “threshold voltage” was found when planar samples of copper were anodized in different electrolytes. For example, for Cu anodized in 0.01 M NaHCO_3_, nanostructures were formed at 15 V and greater voltages, while for copper anodizing at 5 and 10 V, no nanostructures were formed [22]. An analogous situation was found for copper anodizing in 0.1 M Na_2_CO_3_ [12]. Anodization at low voltages, namely 3 and 7 V, did not result in the formation of the nanostructures, although an oxide was obtained.

Thus, the formation of nanostructured anodic oxides on copper foam provides an incredibly high surface area that is suitable for catalytic applications. Accordingly, based on the structural investigation, the formed copper oxides could be suitable for removing the extreme quantity of organic waste such as MB from the wastewater assisted by the UV irradiation process. The absorption spectra of MB using various types of porous copper oxides, anodized at 5 V or 25 V, as a catalyst at various UV irradiation times (up to 380 min) are shown in Supplementary Materials, Figure S1a,b, respectively. In all absorbance-wavelength curves of the MB, a presence of the main distinct peak located at λ equals 660 nm was observed, which is characterizing the absorbance in the MB dye. It can be seen that MB decolorization was enhanced, or the absorbance was decreased with the increasing duration of the UV irradiation with the presence of various copper oxide nanostructures, which means the continuous decrease in MB dye concentration. The maximum decolorization was observed for samples anodized at 25 V, 5 V, and 20 V. The observed reduction in the absorbance with the UV irradiation time confirms the efficient assistance of copper oxide nanoparticles and nanowires as catalysts for degrading the MB. Figure S1c shows the rate of MB dye photocatalytic decolorization versus irradiation time for all copper oxide nanostructures with the copper oxide nanowires formed at 25, 5, and 20 V, showing the highest photocatalytic activity compared to the other samples. The lower photocatalytic activity was observed for samples anodized at 10 V, and this could be due to the smaller surface area of nanoparticles that would limit its absorption in the visible and UV regions.

The photocatalytic decolorization or the removal efficiency (η) of the MB debasement is similar to other materials such as potassium permanganate, methyl orange, etc., and it can be evaluated from the following equation [30]:

(1) $$\eta \;\left(\%\right)=\frac{C_{o}-C}{C_{o}}\times 100=\frac{A_{o}-A}{A_{o}}\times 100$$

where *C_o_* is the initial concentration of waste, *C* is the residual concentration of waste after a fixed irradiation time (*t*), *A_o_* is the initial absorbance of waste, and *A* is the absorbance of waste after a fixed irradiation time. The relation between *A* and *C* is given by the Bouguer–Lambert–Beer equation: *A = εbC*, where *ε* is the molar absorptivity, and *b* is the path length of the sample (b = 1 cm). 

**Figure 5:**
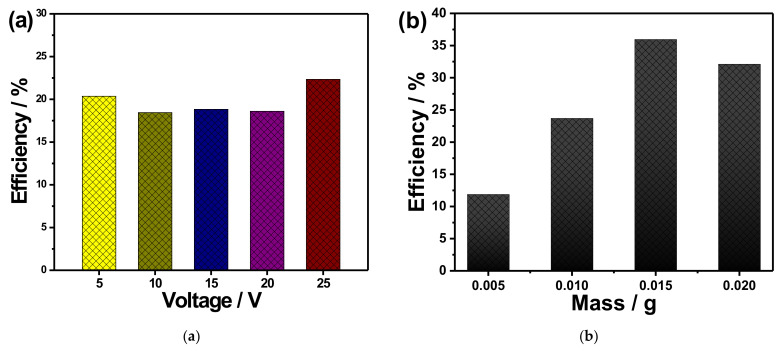
(**a**) Dye decolorization efficiency of MB after irradiation for 380 min, using anodized copper foams as a catalyst (anodized at various anodizing voltages), and (**b**) dye decolorization efficiency of MB after irradiation for 100 min, using various loaded masses of anodized copper foams at 25 V.

Figure 5a shows the relation between *η* and *t* for the photocatalytic decolorization of the MB using anodized copper foams at various anodizing voltages as a catalyst after 380 min. The maximum photocatalytic performance for the decolorization of MB was observed for samples anodized at 25 V, reaching 22.5%, while the minimum efficiency was observed for samples anodized at 10 V with 18.3% efficiency. The small observed values *η* could be attributed to the small amount of copper oxide as catalyst, where the efficiency depends on the catalyst mass. The morphology, optical band gap, amount of catalyst, and other parameters could control the photocatalytic performance of similar systems. For example, systems containing titanium dioxide, either as a powder or immobilized onto the original sample, show much higher *η* values [31,32,33,34] since titanium dioxide is the commercially most acceptable photocatalyst due to its inherent optical and electrical properties and chemical stability. However, the low values *η* noted for this system shouldn’t be discouraging since an improvement in photocatalytic efficiency has been detected. This is due to the fact that even though there is clear assistance of copper oxide nanoparticles and nanowires as catalysts for degrading the MB, the relatively low efficiency proves that the photodegradation process is mostly relying on the samples’ morphologies; however, this requires further investigation.

The influence of mass on the degradation efficiency of MB using anodized copper is highly needed. For this purpose, the measurements were performed using various loading masses (5–20 mg) of copper foam anodized at 25 V for 100 min, and the results are summarized in Figure 5b. Interestingly, the value of efficiency was increased from 12 to 36% as the loaded catalyst increased from 5 to 15 mg, respectively. Meanwhile, a further increase in the amount of the loaded mass leads to a reduction in the degradation efficiencies.

Increasing the irradiation time above 380 min doesn’t affect the efficiency value. In addition, to check the reusability of the investigated catalyst, the efficiency was measured again for anodized copper foam (performed at 25 V) after it was dried. The evaluated efficiency (and after irradiation for 380 min) was 20.3% compared to 22.3% for the virgin sample. The percentage change in evaluated efficiencies is less than 9%.

The adsorbed masses of MB per unit mass of copper oxide nanostructures (*q* in mg/g) at any irradiation time and equilibrium were evaluated from the following equations, respectively [35]:

(2) $$q_{t}=\left(C_{o}-C\right)\frac{V}{W}$$

(3) $$q_{e}=\left(C_{o}-C_{e}\right)\frac{V}{W}$$

where *q_t_* is MB quantities adsorbed capacity at irradiation time (*t*), *q_e_* is equilibrium MB quantities adsorbed, *C_o_* is the initial concentration of MB solution (mg/L), *C* is adsorbate concentrations (mg/L) at a time (*t*), C_e_ is equilibrium adsorbate concentrations (mg/L), *V* is adsorbate volume of MB solution (L), and *W* adsorbent mass (g). Consequently, the dependence of the adsorption capacity of MB on the irradiation time using various copper oxide catalysts is shown in Figure S2. It was observed that there is a significant enhancement of the adsorption capacity as a function of irradiation time using copper oxide anodized at 25 V compared to the other investigated copper oxide nanostructures. The trend of the adsorption capacity is quite similar to the photocatalytic decolorization efficiency curve as a function of the UV irradiation time. In other words, this behavior confirms the increase of the computed photocatalytic efficiency presented in Figure 5 and agrees with other previous studies using other types of catalysts [17,18].

It is important to simulate the photocatalytic data using various kinetic models. Numerous models can be used for analyzing the kinetics of the photocatalytic decolorization experimental data called pseudo-first-order, pseudo-second-order, and intra-particle diffusion kinetic models and can be described by the following relations, respectively [35]:

(4) $$\log\left(q_{e}-q_{t}\right)=\log\left(q_{e}\right)-K_{1}\frac{t}{2.303}$$

(5) $$\frac{t}{q_{t}}=\frac{1}{K_{2}q_{e}^{2}}+\frac{t}{q_{e}}$$

(6) $$q_{t}=K_{diff}\sqrt{t}+C_{k}$$

where *K_1_* (min^–1^) and *K_2_* (g/mg.min) are the pseudo-first and second-order constants, respectively, *K_diff._* is intraparticle diffusion kinetic model (mg/min^1/2^g), and *C_k_* (mg/g) is a kinetic parameter constant that provides an idea of the boundary-layer thickness [36].

**Figure 6:**
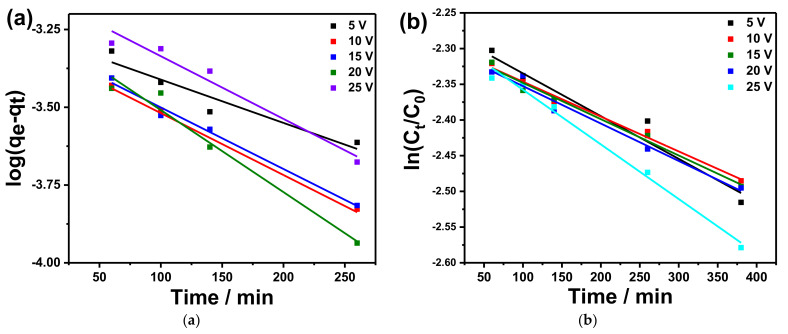
(**a**) Pseudo-first-order kinetic model for adsorption of methylene blue (MB) using anodized copper foam in 0.1 M K_2_CO_3_ at 5, 10, 15, 20, 25, and 25 V as a catalyst; (**b**) the linear relation of ln (*C*/*C_o_*) versus the illumination time of MB in the presence of various anodized copper foam in 0.1 M K_2_CO_3_ at 5, 10, 15, 20, 25, and 25 V as a catalyst.

The experimental data of MB adsorption kinetic onto the surface of various copper oxide nanostructures were investigated using the mentioned previous models. The relations shown by Equations (4)–(6) are demonstrated in Figure 6a, Figures S3 and S4, respectively. The value of various kinetic parameters *K_1_*, *K_2_,* and *K_diff_* was calculated and summarized in Table 2. These values, as well as the values of experimental q_max_ values, are listed in Table 2.

Figure 6a shows the pseudo-first-order kinetic model for MB adsorption on the copper oxide nanostructures with a clear linear relation between $\log\left(q_{e}-q_{t}\right)$ and *t*, which is in agreement with Equation (4), and accordingly, the values of *K_1_* can be determined from the slope of the fitted lines. The fitting correlation coefficients (R^2^) for the applied pseudo-first-order kinetic model for copper foams anodized at 5, 10, 15, 20, and 25 V are equal to 0.94, 0.99, 0.99, 0.97, and 0.90, respectively. The minimum estimated value of *K_1_* (3.2 × 10^−3^ min^−1^) was observed for copper foams anodized at 5 V with *q_e_* of 5.17 × 10^−4^ mg/g, while the maximum value of *K_I_* (6.08 × 10^−3^ min^−1^) was observed for copper foams anodized at 20 V with *q_e_* of 4.71 × 10^−4^ mg/g. In this work, it has been shown that the adsorption of MB on copper oxide nanostructures has been conducted by a single step. These results are in agreement with results reported in the literature [20].

To investigate the photocatalytic activity of copper oxide nanostructures, the decolorization rate constants of MB debasement were ascertained using the well-known Langmuir–Hinshelwood kinetics. The disintegration rate of a pseudo-first-order response is given from the following equation [36]:

(7) $$\ln\left(\frac{C}{C_{0}}\right)=-k_{app}t$$

where *C_o_* is the initial concentration, *C* is the temporal concentration, *t* is the corresponding reaction time, and *k_app_* is the apparent rate constant. Figure 6b shows the plot of ln(*C*/*C_o_*) versus the irradiation time (*t*). The relation between ln(*C*/*C_o_*) and *t* is given by a straight line with a good fitting that well agrees with Equation (7). The fitting correlation coefficients (R^2^) for the kinetic curves of reactions for copper foams anodized at 5, 10, 15, 20, and 25 V are equal to 0.98, 0.99, 0.98, 0.98, and 0.95, respectively. This observation confirms that the MB waste followed a fundamentally pseudo-first-order kinetic model. The determined value of *k_app_* was deduced from the slope of the fitted line and varies from 4.88 × 10^−4^/min for copper foams anodized at 20 V to 7.65 × 10^−4^/min for copper foams anodized at 10 V. It can be concluded that the copper oxide nanowires are an auxiliary material that can enhance the photocatalytic effect but not the active material which is in good agreement with other work [22].

The sample morphology is most likely of greater influence than the composition in this case, considering the similar degradation efficiency among all the samples. Additionally, the morphology of the first sample (5 V) is most likely rich in remnants of the original surface, and that could explain its efficiency. At the same time, the morphology of the last sample (25 V) has some definite nanostructures allowing a higher surface area suitable for photocatalysis.

## 4. Conclusions

Anodization of copper foam in 0.1 M K_2_CO_3_ resulted in the formation of the oxides with developed surface area. The research shows that copper structured or textured elements with strongly developed surface areas, such as foams, can be anodized successfully. The obtained oxides consisted of various crystalline phases like cuprite, Cu_2_O, tenorite, CuO, paramelconite, Cu_4_O_3_, and spertiniite, Cu(OH)_2_. The oxide also possessed malachite, Cu_2_CO_3_(OH)_2_, that confirmed the incorporation of electrolyte anions into the grown oxide. What is important, anodization at 20 and 25 V allowed the formation of nanowires on the copper foam, resulting in high surface area, which encouraged the authors to catalytic research. The highest photocatalytic activity was noted for samples anodized at 25 V, 5 V, and 20 V; however, those values are not substantial. This could be due to the morphology of the samples, the small amount of copper oxide consumed as a catalyst, the optical bandgap of the material, etc. Nevertheless, an improvement in photocatalytic effectiveness has been observed. In this paper, it has been shown that the adsorption of MB on copper oxide has been conducted through a single step and that the MB waste followed a pseudo-first-order kinetic model.

## Figures and Tables

**Table 1:** The crystal structure, interplanar spacing (d), average crystallite size (D), microstrain (ε), and dislocation density (δ) for various formed phases.

Phase	Crystal System	d (Å)	D (nm)					ε × 10^−2^					δ × 10^−3^ (nm^–2^)					JCPDS card
			5 V	10 V	15 V	20 V	25 V	5 V	10 V	15 V	20 V	25 V	5 V	10 V	15 V	20 V	25 V	
Cu(OH)_2_	Orthorhombic	3.73	24.01	20.01	26.67	24.28	25.99	2.9	3.5	2.6	2.9	2.7	1.7	2.5	1.4	1.7	1.5	00-013-0420
Cu_2_O	Cubic	3.02	14.53	23.78	27.24	25.65	30.32	3.9	2.4	2.1	2.2	1.9	4.7	1.8	1.3	1.5	1.1	01-078-2076
Cu_4_O_3_	Tetragonal	2.88	33.71	17.62	21.77	23.99	19.84	1.6	3.1	2.5	2.3	2.7	0.9	3.2	2.1	1.7	2.5	01-083-1665
Cu_2_O_3_(OH)_2_	Monoclinic	2.82	25.08	24.58	17.78	23.74	19.98	2.1	2.1	3.0	2.2	2.6	1.6	1.7	3.2	1.8	2.5	01-076-0660
CuO	Monoclinic	2.53	10.91	11.53	19.05	29.78	25.33	4.4	4.1	2.5	1.6	1.9	08.4	7.5	2.8	1.1	1.6	00-048-1548
Cu_2_O	Cubic	2.46	20.69	18.52	20.11	24.19	24.96	2.3	2.5	2.3	1.9	1.9	2.3	2.9	2.5	1.7	1.6	01-078-2076
Cu_2_O	Cubic	2.13	21.69	18.35	26.97	30.38	28.99	1.9	2.2	1.5	1.3	1.4	2.1	3.0	1.4	1.1	1.2	01-078-2076
Cu_2_O_3_(OH)_2_	Monoclinic	2.10	31.47	30.22	34.57	23.26	21.77	1.3	1.3	1.1	1.7	1.4	1.0	1.1	0.8	1.8	2.1	01-076-0660
Cu	Cubic	1.81	25.61	23.02	31.00	27.03	14.14	1.3	1.5	1.1	1.3	2.4	1.5	1.9	1.0	1.4	5.0	00-001-1241
Cu_2_O	Cubic	1.51	17.74	18.80	14.02	18.51	16.31	1.6	1.5	2.0	1.5	1.7	3.2	2.8	5.1	2.9	3.8	01-078-2076
CuO	Monoclinic	1.42	29.86	15.71	19.96	26.11	24.33	0.9	1.7	1.3	1.0	1.1	1.1	4.0	2.5	1.5	1.7	00-048-1548

**Table 2:** Pseudo-first-order, pseudo-second-order parameters, and experimental q_e_ and parameter values of the intra-particle diffusion model for the adsorption of methylene blue using anodized copper foam in 0.1 M K_2_CO_3_ at 5, 10, 15, 20, 25, and 25 V as a catalyst.

Parameter	Anodizing Voltage (V)				
	5	10	15	20	25
$K_{1}\times 10^{-3}$ (min^−1^)	3.20	4.58	4.56	6.08	4.65
$K_{2}$ (g/mg min)	1.54	1.08	‘0.60	0.41	5.79
$k_{diff}\times 10^{-5}$ (mg/min^1/2^g)	2.74	2.69	2.75	3.17	3.72
$k_{app}\times 10^{-4}$ (min^−^^1^)	5.24	7.65	5.09	4.88	5.98
$q_{e}$ $\times 10^{-4}$ (mg/g)	5.17	4.71	4.79	4.71	5.45

## Data Availability

Data are available on demand.

## Supplementary Materials

The following are available online at https://www.mdpi.com/article/10.3390/ma14175030/s1, Figure S1. The optical absorbance spectra of MB at various periods of irradiation to the ultraviolet irradiation using anodized copper foam in 0.1 M K_2_CO_3_ at (a) 5, and (b) 25 V as a catalyst, and (c) rate of MB dye photodegradation with anodized copper foam as catalysts. Figure S2. Histogram plot shows the adsorption capacity (*q_t_*) of methylene blue (MB) using anodized copper foam in 0.1 M K_2_CO_3_ at various anodizing voltages 5, 10, 15, 20, 25, and 25 V as a catalyst after irradiation for 380 min. Figure S3. Pseudo-second-order kinetic model for adsorption of methylene blue (MB) using anodized copper foam in 0.1 M K_2_CO_3_ at 5, 10, 15, 20, 25, and 25 V as a catalyst. Figure S4. Intra-particle diffusion model for adsorption of methylene blue (MB) using anodized copper foam in 0.1 M K_2_CO_3_ at 5, 10, 15, 20, 25, and 25 V as a catalyst.
