# Supplementary material for: Fabrication, Characterization and Photocatalytic Activity of Copper Oxide Nanowires Formed by Anodization of Copper Foams
Source: Materials (Basel). 2021 Sep 2;14(17):5030. doi: 10.3390/ma14175030 (PMC8433699; doi:10.3390/ma14175030)
Supplement: Supplementary file 1 [file materials-14-05030-s001.zip › materials-1346758-supplementary.pdf]

materials

MDPI

Article

Fabrication, Characterization and Photocatalytic Activity of Copper Oxide Nanowires Formed by Anodization of Copper Foams

Alaa M. Abd-Elnaiem [1,2,*], Moustafa A. Abdel-Rahim [1,2], Atta Y. Abdel-Latief [1,2], Ahmed Abdel-Rahim Mohamed [1,2], Kristina Mojsilović [3] and Wojciech Jerzy Stępniowski [4,*]

1 Physics Department, Faculty of Science, Assiut University, Assiut 71516, Egypt; mabdelrahim@aun.edu.eg (M.A.A.-R.); attayousef@aun.edu.eg (A.Y.A.-L.); ahmed.201297@science.au.edu.eg (A.A.-R.M.)
2 Academy of Scientific Research and Technology (ASRT) of the Arab Republic of Egypt, Cairo 11516, Egypt
3 Faculty of Physics, University of Belgrade, Studentski trg 12–16, 11000 Belgrade, Serbia; kristina.mojsilovic@ff.bg.ac.rs
4 Faculty of Advanced Technology and Chemistry, Institute of Materials Science & Engineering, Military University of Technology, Kaliskiego 2 Str., 00908 Warszawa, Poland
* Correspondence: abd-elnaiem@aun.edu.eg (A.M.A.-E.); wojciech.stepniowski@wat.edu.pl (W.J.S.)

Keywords: copper oxides; anodization; nanowires; FTIR; photocatalytic decolorization; nanostructures; tenorite; cuprite; paramelconite; malachite

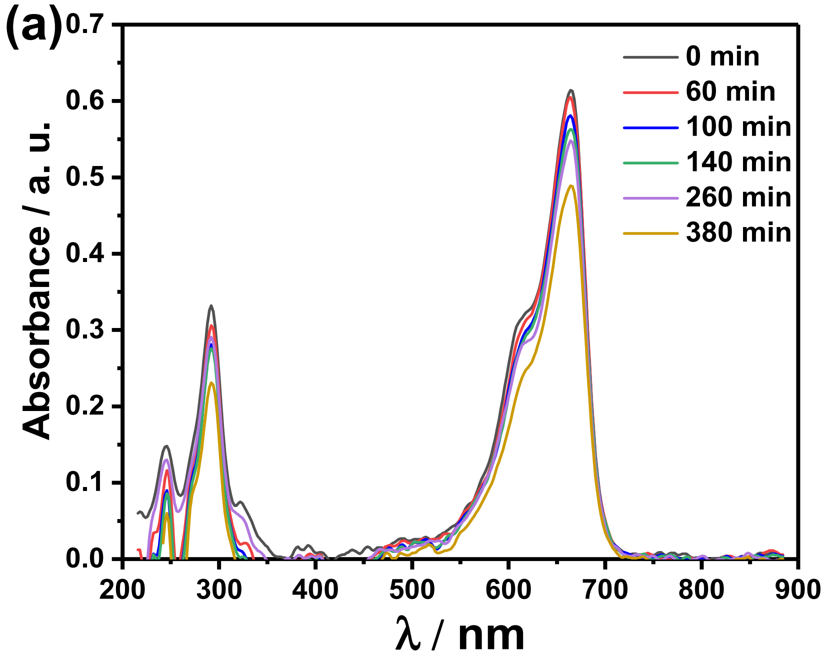

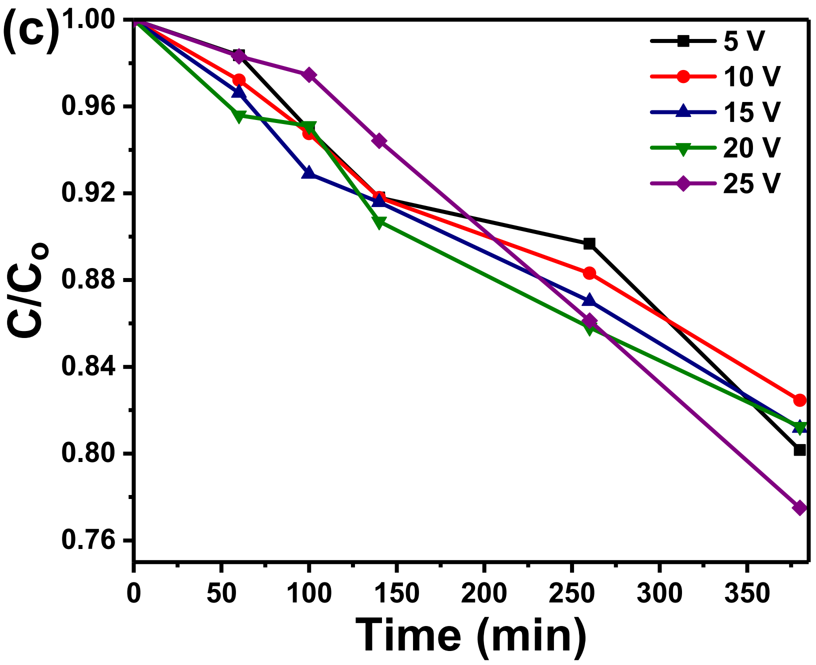

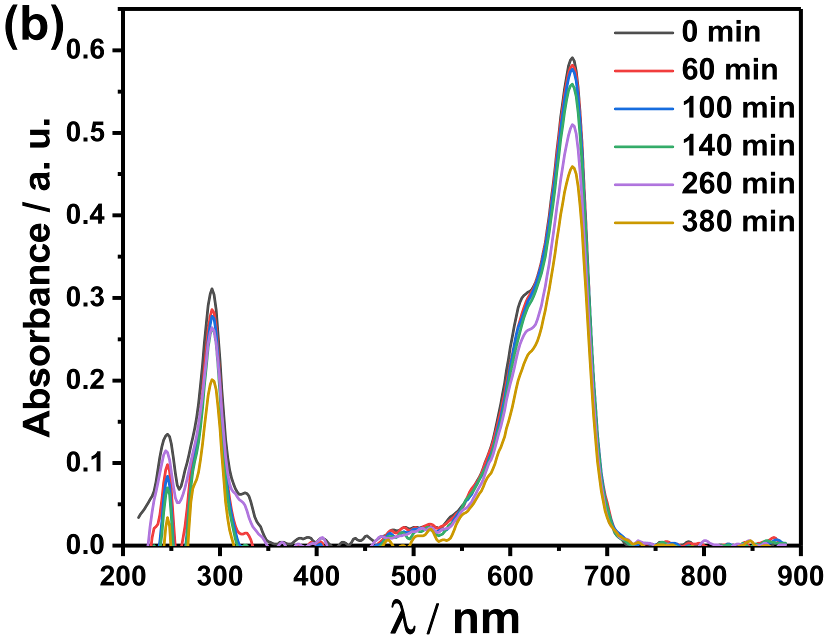

Figure S1. The optical absorbance spectra of MB at various periods of irradiation to the ultraviolet irradiation using anodized copper foam in 0.1 M K_2CO_3 at (**a**) 5, and (**b**) 25 V as a catalyst, and (**c**) rate of MB dye photodegradation with anodized copper foam as catalysts.

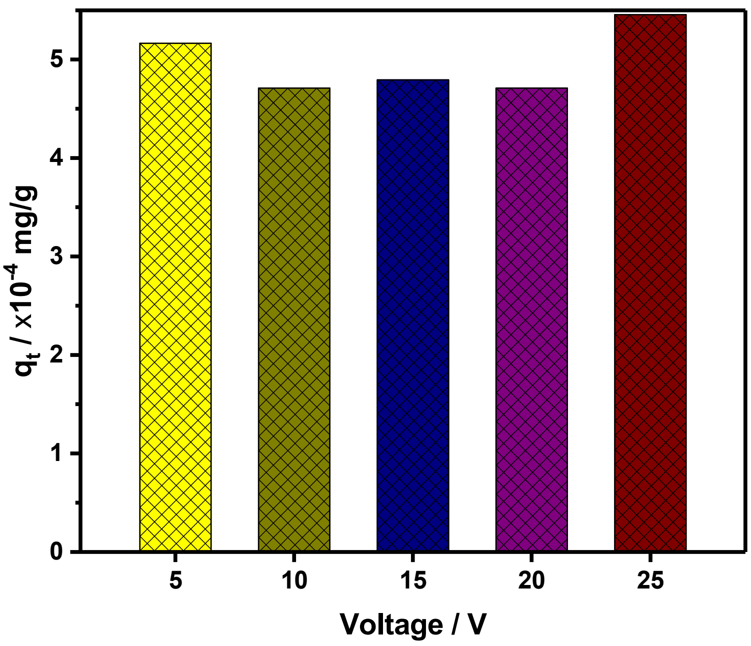

Figure S2. Histogram plot shows the adsorption capacity (q_t) of methylene blue (MB) using anodized copper foam in 0.1 M K_2CO_3 at various anodizing voltages 5, 10, 15, 20, 25, and 25 V as a catalyst after irradiation for 380 min.

Figure S3 illustrates the pseudo-second-order kinetic model for MB degradation on the copper oxide nanostructures. It illustrates a linear plot for the relation of $\frac{t}{q_t}$ versus t. The fitting correlation coefficients (R^2) for the applied pseudo-second-order kinetic for copper foams anodized at 5, 10, 15, 20, and 25 V are equals 0.20, 0.82, 0.58, 0.27, and 0.78, respectively. From the intercept of the fitted lines, the value of K_2 is obtained and changed between 0.41-5.79 g/mg·min and is listed for various copper oxide samples in **Table 1**. The maximum and minimum value of K_2 was for copper foams anodized at 25, and 20 V, respectively. Furthermore, the values of q_e were calculated for MB degradation using the copper oxide nanostructured were calculated from the slope of the fitted line in **Figure S3** and listed in **Table 1**.

The relation between q_t and $\sqrt{t}$, for the degradation process for MB using copper oxide nanostructured is shown in **Figure S4**. It illustrates a linear plot for the relation of q_t and $\sqrt{t}$. The fitting correlation coefficients (R^2) for the applied intra-particle diffusion kinetic model for copper foams anodized at 5, 10, 15, 20, and 25 V are equals 0.92, 0.98, 0.98, 0.94, and 0.95, respectively. From the obtained and fitted line, we have calculated both the values of $K_{diff.}$ and C for copper oxide nanostructures. The maximum estimated values of $K_{diff.}$ and C are 3.72×10^{-5} mg/min$^{1/2}$g, and -2.85×10^{-4}, respectively, and they were observed for adsorption of MB onto copper foams anodized at 25 V. These values for other investigated samples are listed in **Table 1**.

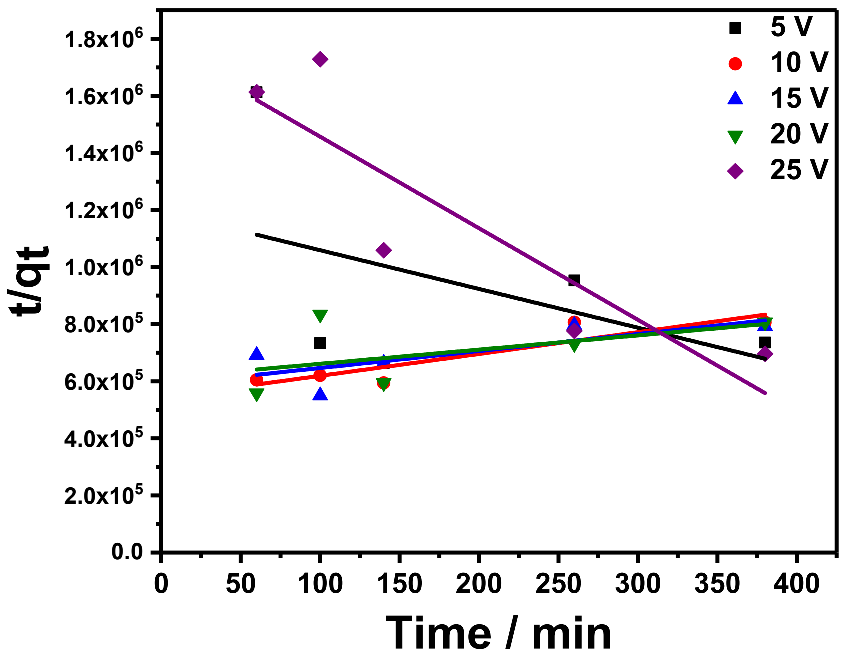

Figure S3. Pseudo-second-order kinetic model for adsorption of methylene blue (MB) using anodized copper foam in 0.1 M K_2CO_3 at 5, 10, 15, 20, 25, and 25 V as a catalyst.

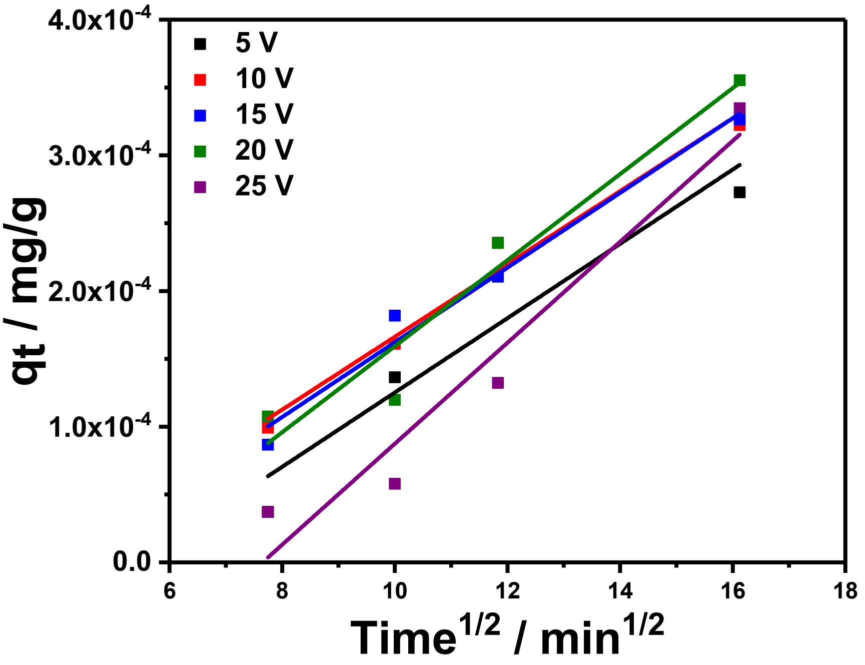

Figure S4. Intra-particle diffusion model for adsorption of methylene blue (MB) using anodized copper foam in 0.1 M K_2CO_3 at 5, 10, 15, 20, 25, and 25 V as a catalyst.
